# Transcriptomics and proteomics association analysis demystify the molecular mechanisms underlying epididymal sperm maturation disorders in yaks with cryptorchidism

**DOI:** 10.1093/jas/skaf397

**Published:** 2025-11-11

**Authors:** Dapeng Yang, Ligang Yuan, Yumei Qi, Haijun Li, Qinghai Ren, Yubao Li

**Affiliations:** Phage Research Center, Liaocheng University, Liaocheng, Shandong 252000, China; Shandong Key Laboratory of Applied Technology for Protein and Peptide Drugs, Liaocheng University, Liaocheng, Shandong 252059, China; College of Veterinary Medicine, Gansu Agricultural University, Lanzhou 730070, China; College of Veterinary Medicine, Gansu Agricultural University, Lanzhou 730070, China; College of Veterinary Medicine, Gansu Agricultural University, Lanzhou 730070, China; Phage Research Center, Liaocheng University, Liaocheng, Shandong 252000, China; Shandong Key Laboratory of Applied Technology for Protein and Peptide Drugs, Liaocheng University, Liaocheng, Shandong 252059, China; Phage Research Center, Liaocheng University, Liaocheng, Shandong 252000, China; Shandong Key Laboratory of Applied Technology for Protein and Peptide Drugs, Liaocheng University, Liaocheng, Shandong 252059, China

**Keywords:** cryptorchidism, ductus epididymidis, proteomics, spermatozoa, transcriptomics, yak

## Abstract

Cryptorchidism is a prevalent reproductive disorder in male mammals. Yaks, being a unique species inhabiting high altitudes, exhibit a higher prevalence of cryptorchidism compared to cattle in lowland areas, leading to a significant constraint on their reproductive efficiency. This study conducted a comprehensive analysis of the pathological characteristics and molecular regulatory mechanisms of epididymal tissue in cryptorchidism yaks through the integration of histological, transcriptomic, and proteomic approaches. The pathological analysis showed that the epididymal wall of cryptorchid yak was significantly atrophic, the lumen was narrow and the sperm was less, accompanied by abnormal deposition of elastic fibers and collagen fibers, reticular fiber degradation, and other structural remodeling. Furthermore, electron microscopy observations indicated folded nuclear membranes in epididymal cells of cryptorchidism yaks, accompanied by chromatin condensation, marginalization, and mitochondrial swelling. Transcriptionomics screened out 287 differentially expressed genes (|log2FC|>1, FDR-adjusted *P* < 0.05). These DEGs were mainly enriched in GO entries such as extracellular matrix reconstruction, signal receptor binding, and steroid synthesis. In addition, KEGG analysis showed that Wnt, PI3K-Akt, tight junction and calcium ion signaling pathways were significantly downregulated in cryptordidymis epididymis. Proteomics identified 114 differential proteins, among which the expression of key Wnt pathway proteins (such as SYNPO and LMCD1) and tight junction proteins (TJP2 and ZO-1) were down-regulated, and lysosomal function-related proteins were significantly activated. Multiomic association analysis revealed that only a few genes were expressed consistently at mRNA and protein levels, indicating that post-translational modification plays an important role in cryptorchidism pathology. The qRT-PCR and Western blot (WB) experiments further confirmed that the differential genes were highly consistent with the expression trend and subcellular localization of the proteins. This study not only provides a potential target for the treatment of cryptorchidism in high altitude areas but also accumulates important data for understanding the regulation of epididymal function and the mechanism of male infertility.

## Teaser

This study explored the molecular mechanism of epididymal sperm processing disorder in cryptorchidism yaks, elucidated the synergistic mechanism of Wnt/tight junction pathway imbalance and lysosomal overactivation, and provided a theoretical basis for the development of targeted therapy intervention strategies. The results of this study not only fill the gap in the study of epididymis in animals with cryptorchidism but also open up a new perspective for the exploration of the pathological mechanism of human cryptorchidism.

## Introduction

The epididymis is a vital component of the reproductive system in male animals. It is located on the dorsal side of the testis and is composed of highly coiled catheters that connect the testicular efferent tubules with the vas deferens. Epididymis is crucial for various functions, including sperm maturation, storage, nourishment, regulation, fertilization, and immune defense. Cryptorchidism is a common congenital reproductive system abnormality in mammals that is characterized by the failure of unilateral or bilateral testes to descend to the scrotum. According to statistics, about 0.17% of bulls worldwide have cryptorchidism, of which 34% of the testes are located in the abdominal cavity (St Jean et al., 1992; Mieusset et al., 1997). The occurrence of cryptorchidism is related to many factors, including genetic, environmental, and endocrine factors. Genetic factors may affect the expression of related genes in the process of testicular descent, leading to cryptorchidism. Abnormal expression of genes, such as NEK2, RXFP2, KAT6, and H3K9, is the cause of cryptorchidism (Ge et al., 2020; Nowacka-Woszuk et al., 2020; Stachowiak et al., 2024). Environmental factors, such as maternal exposure to chemicals, radiation, and oxygen deprivation during pregnancy, negatively affect the natural descent of the testes. Endocrine factors, particularly abnormalities of the endocrine system, especially insufficient secretion of gonadotropins, can also contribute to the development of cryptorchidism. Moreover, the abnormal expression of some proteins in the process of testicular descent is also one of the important causes of cryptorchidism, including INSL-3, NSE, SP, NFH, and DβH, which are closely related to the formation of cryptorchidism (Krausz et al., 2000; Yuan et al., 2021; Yuan et al., 2022).

Recently, the integrated application of transcriptomics and proteomics has provided a new perspective for analyzing the molecular mechanism, diagnostic markers, and therapeutic targets of cryptorchidism. Transcriptomics systematically analyzes differentially expressed genes (DEGs) between cryptorchid testis and normal testis by RNA sequencing (RNA-seq) or microarray technology to reveal key regulatory pathways. For example, in the mouse cryptorchidism model, the expression of testicular descent-related genes insulin-like peptide 3 (INSL3) and homeobox protein Hox-A10 (Hoxa10) was significantly reduced. Among these, INSL3 encodes insulin-like peptide hormones and regulates the development of testicular gubernaculum. INSL3 deletion can lead to the cryptorchidism phenotype (Feng et al., 2006; Ferlin et al., 2008). Furthermore, transcriptome analysis of human cryptorchid testes showed that NF-κB and TNF-α signaling pathways were abnormally activated, suggesting that chronic inflammation and oxidative damage may aggravate germ cell apoptosis (Hadziselimovic et al., 2014). Proteomics identifies differentially expressed proteins (DEPs) in cryptorchidism using mass spectrometry, focusing on post-translational modifications (PTMs) and functional interaction networks. Proteomics studies have shown that downregulation of the expression of key proteins STRA8 and SCP3 in the testicular meiosis of cryptorchidism pigs can lead to spermatogenesis arrest (Yue et al., 2019). Additionally, proteomics studies of various parts of the bovine epididymis have found that proteins, such as clusterin and prosaposin, are involved in biological processes, such as sperm membrane remodeling and motor ability acquisition (Belleannée et al., 2013). At present, most studies on cryptorchidism focus on the testis. Still, there is a lack of attention to the pathological changes and regulatory mechanisms of epididymis in animals with cryptorchidism.

Yak is a unique economic animal in Central Asia and an important source of meat for nomadic people in high-altitude areas of Central Asia. It has important economic and cultural value (Prakash et al., 2008; Wan et al., 2021). Yaks live in the Qinghai–Tibet Plateau area above 3,000 m above sea level all year round. The present study was conducted on the epididymal tissues of yaks with cryptorchidism using comparative proteomic and transcriptomic analyses to further understand the effects of cryptorchidism on the function of the yak epididymis. Transcriptomic and proteomic analyses allowed us to analyze the effects of cryptorchidism on the structure and function of yak epididymis at the molecular level and to reveal the pathological changes and regulatory mechanisms of epididymis caused by cryptorchidism. This study helps to fill the gap in the current research on the epididymis of cryptorchid animals, providing a new perspective for understanding the regulatory mechanism during sperm maturation and storage. This study is of great significance for improving the reproductive efficiency and reproductive health of yaks.

## Materials and Methods

### Animal welfare statement

All experimental procedures were approved by the Animal Care and Use Committee of the Veterinary College of Gansu Agricultural University (Approval No. GSAU-Eth-VMC-2023-005).

### Experimental animals and sample collection

Yak epididymis samples were collected from the Datong pastoral area (average elevation: 3100 m) in Qinghai Province, China, during the breeding season (August-September). Twenty adult male yaks aged 3–4 yr were used in this experiment, including 10 yaks with cryptorchidism (all were unilateral cryptorchidism). Epididymal tissue was resected by surgery. Three pathological samples were divided equally and stored in 4% paraformaldehyde solution and 2.5% glutaraldehyde solution, respectively, for histochemical and ultrastructural studies. The remaining pathological samples were taken out after rapid freezing in liquid nitrogen. Four were stored in a refrigerator at −80°C for RNA and protein extraction, and three were stored in dry ice and sent to Chengdu Nuomei Metabolic Biotechnology Co., Ltd. for testing. The sample processing method of the control group (epididymis of normal yak) was the same as that of the pathological group. The samples used for transcriptome and proteomics sequencing were the same.

### Histological sample preparation and observation

The yak epididymis samples preserved in 4% paraformaldehyde solution were taken out and prepared into tissue sections by conventional dehydration, waxing, and paraffin embedding, which were used for the histochemical study and pathological section staining, respectively.

Hematoxylin–Eosin (H & E) staining: Tissue sections were stained with hematoxylin and then re-stained with eosin, dehydrated with gradient alcohol and then sealed, observed and photographed under an ordinary light microscope. Elastic fibers (EF), collagen fibers (CF) and reticular fibers (RF) were stained using a staining kit, and the staining steps were performed according to the kit instructions (G1597, G1473, and G3535, Solarbio Technology Co., Beijing, China).

Transmission electron microscopy sample preparation steps were as follows. The yak epididymis sample was placed in a 2.5% glutaraldehyde solution for fixation. After 1 wk of fixation, the sample was taken out and cut into small cubes of 0.2 cm × 0.2 cm × 0.2 cm and fixed with 2% osmium tetroxide solution at 4°C for 3 h. Subsequently, the samples were dehydrated using a gradient acetone series (30%, 50%, 70%, 80%, 90%, 95%, 100%) and embedded in epoxy resin. After the samples were made into ultrathin sections, they were fixed on a copper mesh and stained with uranyl acetate and lead citrate. Finally, they were observed and photographed using a JEM-100CX electron microscope (Japan NEC).

### RNA extraction and transcriptomics

According to the instructions of the kit, total RNA was extracted from each epididymis sample using Trizol Reagent (R1100, Solarbio, Beijing, China). The quality and concentration of the RNA were evaluated on a NanoDrop spectrophotometer (Thermo Fisher, Waltham, MA, USA), an Agilent 2100 Bioanalyzer (Agilent Technologies, Santa Clara, CA, USA), and an Ultramicro Nucleic Acid/Protein Quantifier (Shanghai Aoran Science and Trade Co., Shanghai, China). PolyA-selected mRNA was fragmented (200–300 bp) using magnesium ions. Double-stranded cDNA was synthesized from fragmented mRNA using random hexamers, and PCR amplified to 300–400 bp. Libraries were quality controlled (Agilent 2100) and quantified by fluorescence before Illumina HiSeq paired-end sequencing. DEGs were identified using the screening criteria of |Log2FC| > 1 and FDR-corrected *P*-value < 0.05, followed by functional annotation via gene set enrichment analysis (GSEA) (Ding et al., 2023; Chen et al., 2024; Teng et al., 2024a).

### RT-qPCR validation

The extracted RNA was reverse-transcribed into cDNA using a reverse transcription kit (AT311-02, Beijing TransGen Biotech Co., Ltd, Beijing, China) and stored for later use. Primers were designed using Primer Premier 5.0 (Biosoft International, Palo Alto, USA) based on the NCBI database (www.ncbi.nlm.nih.gov) and were synthesized by Shanghai Sangon Biotechnology Co., Ltd (Table 1). The bovine β-actin gene was used as an internal reference. The cDNA was diluted to a concentration of 200–300 ng/mL, while all the primers were diluted to a concentration of 10 μmol/L. qPCR was performed on a Light Cycler 480 (Roche, Germany). The cycling conditions were 95°C for 30 s, followed by 45 cycles of 95°C for 5 s, 50–60°C for 15 s, and 72°C for 10 s (Yang et al., 2022). At least three replicates were run for each sample to ensure the accuracy of the results.

**Table 1.** List of primers used for validation of the DEGs in the yak epididymis

Gene name	Abbreviations	Sequences (5'→3')	Length (bp)	Tm (°C)	Primer efficiency (%)	Accession no.
**Signal transducing adaptor family member 1**	STAP1	CTGAACCCTATGCCAGCATGT	136	58	98	XM_005892889
		GCCGGATGGTGATGGAGTAA				
**G protein-coupled receptor 68**	GPR68	TTCCACCAGTTCCGCACTTT	184	60	95	XM_005892611.2
		AGCGGTAGTAGTTGATGCCG				
**ROS proto-oncogene 1**	ROS1	GCTGAACGAACCCCAGTACA	195	60	100	XM_005900060.2
		GCTGCCAGATCCCTGTGAAT				
**Calbindin 1**	CALB1	CATTTCGACGCTGACGGAAG	189	59	96	XM_005888294.2
		TGTGGGTAACACATGAGCCAA				
**Early growth response 2**	EGR2	GTCGGTGACCATTTTCCCCA	81	59	99	XM_005896713.2
		TTGATCATGCCATCTCCGGC				
**Adrenoceptor alpha 1A**	ADRA1A	GTGATGCCCATTGGGTCTTTC	195	60	101	XM_005903000.2
		GGTGTGTTTGGAGGACTGCT				
**Fibroblast growth factor 10**	FGF10	GCATGTGCGGAGCTACAATC	140	59	98	XM_005894857.2
		TCTCCAGGATACTGTACGGG				
**Endothelin receptor type A**	EDNRA	GCATCTGAAACAGCGTCGAG	139	58	99	XM_005909847.1
		TTCACATCGGTTCGTGTCCA				
**Carnosine dipeptidase 1**	CNDP1	CTGGTCGCTCTTCTTGGCAG	117	59	101	XM_005904305.2
		GTCGATGGCCTCGTATACCC				
**Transcription and immune response regulators**	TCIM	GCTGTTGCCTCGTACGTTTC	175	60	99	XM_005897526.2
		ATCAGCGCCAGTCTTTCTCC				
**Actin, cytoplasmic 1**	ACTB	ACGGTGCCCATCTACGAGG	153	60	102	DQ838049.1
		CTTGATGTCACGGACGATTT				

### Total protein extraction

Epididymal samples were ground to a powder in liquid nitrogen, mixed with lysis buffer containing a protease inhibitor (lysate: protease inhibitor 50:1), vortexed, ultrasonicated for 1 s, stopped for 1 s, and ultrasonicated again for 5 min. The samples were then centrifuged at 14,000 rpm for 20 min. A 10-μL aliquot was used for quantification using the Bradford method, and the rest was stored at −80°C.

### Liquid chromatography-tandem mass spectrometry (LC-MS/MS)

The mobile phase was 0.1% formic acid in water (solvent A), 80% acetonitrile, 0.1% formic acid, and 20% water (solvent B). The ground lyophilized powder sample was dissolved in 10 μL of solvent A and centrifuged at 14,000 rpm for 20 min at 4°C. A 1-μL volume of the sample supernatant was injected into the chromatographic column for separation. An Orbitrap Fusion Lumos mass spectrometer equipped with a nanospray ion source (Nanospray Flex, Thermo Fischer Scientific) was used for mass spectrometry. The spectrometer was operated in data-dependent acquisition mode; data were acquired in full-scan mode over a range of 300–1,500 m/z at a resolution of 120,000 (200 m/z), with the automatic gain control (AGC) target set at 5 × 105 and the C-trap maximum injection time set to 50 ms. Secondary ions were detected using Orbitrap at a resolution of 50,000 (200 m/z). The AGC target was set at 1 × 105, the maximum injection time was set at 100 ms, and the collision energy of the peptide fragmentation was set to 38%.

### Verification using WB

Yak cryptorchid and normal epididymal tissues previously stored at −80°C were ground to a fine powder using a freezing grinder. Lysis buffer (1 mL, Cat: R0010, Solarbio Biotechnology Co., Ltd, Beijing, China) containing 1 μL of phosphatase inhibitor and 1 μL of phenylmethanesulphonyl fluoride (PMSF) was added to each sample, followed by five rounds of shaking in an ice box at low temperature for 10 min each time. The samples were centrifuged at 4 °C at 12,000 rmp for 15 min using a low-temperature centrifuge, and the supernatant was stored at −80°C. The extracted protein was quantified using a bicinchoninic Acid (BCA) protein quantification kit (PC0020, Solarbio Biotechnology Co., Ltd, Beijing, China). Each sample was diluted to the same concentration. After denaturation and electrophoresis, the protein was transferred to a PVDF membrane (Cat: YA1701, Solarbio Biotechnology Co., Ltd, Beijing, China), blocked with skimmed milk powder and incubated with primary antibody (Antibody information is provided in Table 2) at 37°C for 2 h. After washing five times with Tris-buffered saline + Tween 20 (TBST), 10 min each wash, the membrane was incubated with the secondary antibody (diluted 1:3000) at 37°C for 1.5 h and washed five times with TBST for 10 min each time. An ECL (Cat: PE0010, Solarbio Biotechnology Co., Ltd, Beijing, China) chemiluminescence reagent was added dropwise, and the signal was observed under a chemiluminescence instrument (Teng et al., 2024b).

**Table 2.** Immunofluorescence and WB primary antibody information table

Antibody name	Manufacturer	Product code	Dilution ratio
**ME1**	Beijing Bioss Biotechnology Co., Ltd.	bs-5084R	WB:1:300
**PLOD3**	Beijing Bioss Biotechnology Co., Ltd.	bs-12732R	WB:1:300
**RBMY**	Beijing Bioss Biotechnology Co., Ltd.	bs-12290R	WB:1:300
**TMEM134**	Beijing Bioss Biotechnology Co., Ltd.	bs-19954P	WB:1:300
**PUS7L**	Beijing Bioss Biotechnology Co., Ltd.	bs-21112R	WB:1:300
**SYNPO**	Jiangsu Qinke Biological Research Centre Co., Ltd.	DF12173	IF:1:300 WB:1:400
**SYNPO2**	Beijing Bioss Biotechnology Co., Ltd.	bs-8743R	IF:1:300
**TJP2**	Jiangsu Qinke Biological Research Centre Co., Ltd.	DF8632	IF:1:500
**ZO-1**	Jiangsu Qinke Biological Research Centre Co., Ltd.	AF5145	IF:1:300
**LMCD1**	Jiangsu Qinke Biological Research Centre Co., Ltd.	DF15126	IF:1:500
**MCU**	Jiangsu Qinke Biological Research Centre Co., Ltd.	DF9403	IF:1:500
**β-actin**	Jiangsu Qinke Biological Research Centre Co., Ltd.	AF7018	IF:1:800

### Immunofluorescence

Previously fixed yak cryptorchid and normal epididymal tissues were washed in running water for 24 h, dehydrated through a graded alcohol series, paraffin embedded, and sectioned. The paraffin sections were routinely dewaxed with xylene and rehydrated through a decreasing alcohol gradient. Antigen repair was performed in a high-pressure cooker for 15 min. After cooling to room temperature, 3% H_2_O_2_ in deionized water was added, followed by incubation at 37°C for 15 min to eliminate endogenous peroxidase activity. After washing with PBS, the sections were blocked with a goat serum working solution (added dropwise) and incubated at 37°C for 20 min. Tissue surface reagents were not washed off. The sections were then incubated with rabbit polyclonal antibody (50 μL, Antibody information is provided in Table 2) or 0.01 mol/L PBS (negative control) overnight at 4 °C and washed five times with PBS for 5 min each time. The subsequent steps were performed shielded from light. The first secondary antibody (goat anti-rabbit IgG [H + L] Alexa Fluor 488, 1:1,000, ab150077, Abcam, UK) was added dropwise, followed by incubation for 1 h at 37°C and rinsing five times with PBS for 5 min each time. Subsequently, the sections were incubated first with the second antibody, added dropwise, at 37°C for 4 h, rinsed five times with PBS for 5 min each time, and then with the second secondary antibody (goat anti-rabbit IgG [H + L] Alexa Fluor 647, 1:1,000, ab150115, Abcam, UK). Finally, DAPI (1:800, ab104139, Abcam, UK) was added dropwise, followed by incubation for 1 h. After rinsing with PBS, the slides were sealed and examined under a laser-scanning confocal microscope.

### Data processing

WB data were quantified using ImageJ software. Relative gene expression was calculated using the 2^−ΔΔCT^ method. The obtained data were analyzed with SPSS 17.0 (IBM Technology, Chicago, IL, USA). A *t*-test was used for intergroup comparisons. Histograms were generated using GraphPad 9.0 (v9.0, GraphPad Software Inc., San Diego, CA, USA). Fluorescence intensity was quantified using the Image-Pro Plus image analysis system (v6.0, Media Cybernetics, USA).

## Results

### Histopathological analysis of the yaks epididymis with cryptorchidism

**Figure 1.**
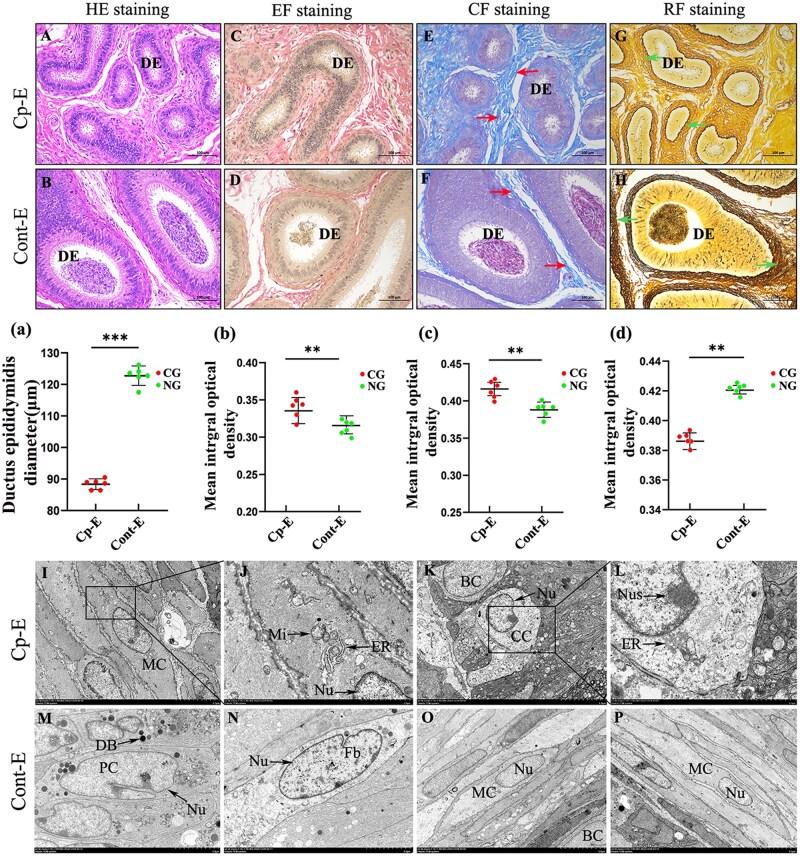
Histological analysis of cryptorchidism and the normal epididymis of yak. (A, B) H & E staining of cryptorchidism and normal epididymis, respectively. (C, D) Elastic fiber staining of cryptorchidism and normal epididymis, respectively. (E, F) Collagen fiber staining of cryptorchidism and normal epididymis, respectively. (G, H) Reticular fiber staining of cryptorchidism and normal epididymis, respectively. (I-P)Transmission electron microscopy images of cryptorchidism and normal epididymis. (a) Comparison of the diameter of the seminiferous tubules in cryptorchidism and normal epididymis. (b–d) Comparison of image integral optical density values. BC: basal cell; CC: clear cells; CG: Cryptorchidism group; DB: dense body; DE: ductus epididymidis; ER: endoplasmic reticulum; Fb: fibroblast; MC: myoid cell; NG: normal group; Nu: Nucleus; Nus: nucleolus; PC: principal cell; Mi: mitochondrion. Cp_E: cryptorchid epididymal tissue; Cont–E: normal epididymal tissue. The red arrows in the diagram indicate collagen fibers, while the green arrows denote reticular fibers. **P < 0.01.

The histopathological analysis of the epididymis of cryptorchidism yak showed significant histological differences between cryptorchidism and the normal control group. H & E staining microscopic observation showed that the epididymal tissue of cryptorchidism yaks showed severe atrophy pathological features, which were manifested by thinning of the epididymal duct wall and significantly reduced lumen diameter compared with the normal control group (*P *< 0.01; Figure 1A, B, a). Notably, densely arranged mature spermatozoa were observed in the lumen of the epididymis in the normal group. In contrast, few spermatozoa were seen in the lumen of the epididymis in the cryptorchidism group (Figure 1A and B). Further analysis of extracellular matrix (ECM) components using special staining techniques revealed that EF staining showed that the distribution density of elastic fibers (black-colored areas) in the interstitial tissue of the cryptorchidism group was significantly higher than that of the control group (Figure 1C and D, b). CF staining quantitative analysis showed that the content of collagen fibers (dark blue area) in the cryptorchidism group was significantly higher than that in the control group (Figure 1E and F, c). The results of the RF staining showed the opposite trend. The distribution density of reticular fibers (black reticular structure) in the control group was significantly higher than that in the cryptorchidism group (Figure 1G and H, d).

The ultrastructure of the epididymis was observed by transmission electron microscopy. The results showed that the ultrastructure of the epididymis in the cryptorchidism group showed significant abnormalities. There was a large amount of collagen fiber deposition in the epididymis of the cryptorchidism group, and the staining was deep. The nuclear morphology of various types of cells was irregular, the nuclear membrane was wrinkled, and chromatin condensation was marginalized, suggesting that the nuclear function was abnormal (Figure 1I–L). The mitochondria of the epididymal duct epithelial cells in the cryptorchidism group were swollen, the ridge structure was blurred, and some vacuoles appeared compared with the normal control group. These results indicate that the energy metabolism function was impaired. The epididymal principal cells in the normal control group secrete a large number of dense bodies. In contrast, the cryptorchidism group secretes a decreased amount of dense bodies (Figure 1M–P).

### Transcriptomics sample quality detection and differential gene screening

**Figure 2.**
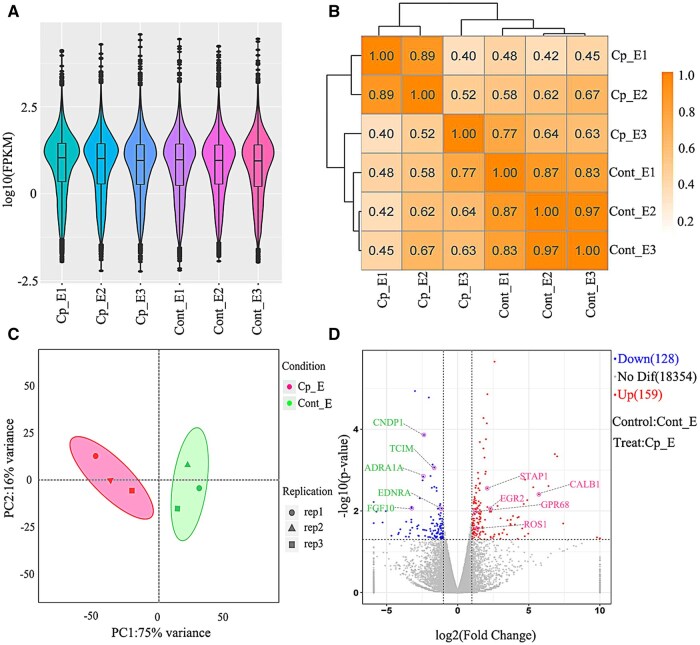
Quality control analysis and differential gene screening of RNA-seq samples. (A) Violin plot of FPKM values in each sample; (B) Sample correlation analysis diagram; (C) Sample principal component analysis diagram; (D) DEG volcano map, with red dots representing upregulated genes, blue dots representing downregulated genes, and gray dots representing non-significant differentially expressed genes. Cp_E: cryptorchid epididymal tissue; Cont_E: normal epididymal tissue.

Based on fragments per kilobase million (FPKM) standardized gene expression analysis, the expression profiles between samples in each experimental group showed a high degree of uniformity, and there was no significant statistical difference in the total gene expression level within the group (Figure 2A). The Pearson correlation coefficient analysis between samples further confirmed that the same type of epididymal tissue samples had excellent biological repeatability (Figure 2B). Principal component analysis (PCA) was performed using the DESeq2 package (R 4.1.2, Canada) in the R language. The results of PCA showed that the samples of different treatment groups showed a significant separation trend in the two-dimensional principal component space. In contrast, the samples in the group were clustered closely (cumulative explained variance > 70%, Figure 2C). This result and the above expression consistency analysis jointly verified the reliability of the experimental design. The DESeq2 algorithm was used to screen DEGs. Significance threshold |log2FoldChange| > 1 and *P*-value < 0.05 corrected by Benjamini-Hochberg. Plot a volcano plot using the ggplot2 package (version 3.4.0, USA) in the R programming language. A total of 287 DEGs were identified by statistical analysis, among which 159 (55.4%) upregulated genes and 128 (44.6%) downregulated genes were specific to the cryptorchidism group (Figure 2D, Supplementary Tables S1–S2).

Based on systematic functional analysis of transcriptome data of cryptorchidism yak epididymis and normal yak epididymis, in this study, gene ontology (GO), Kyoto encyclopedia of genes and genomes (KEGG), and GSEA were integrated to systematically evaluate the biological function of DEGs. Perform GO and KEGG analyses using the KOBAS tool (http://kobas.cbi.pku.edu.cn/), and generate GO bubble charts and bar charts using GraphPad Prism 9. By setting the significance threshold (*P *< 0.01), a total of 276 significantly enriched functional items were selected by GO enrichment analysis, and their classification characteristics were as follows.

**Figure 3.**
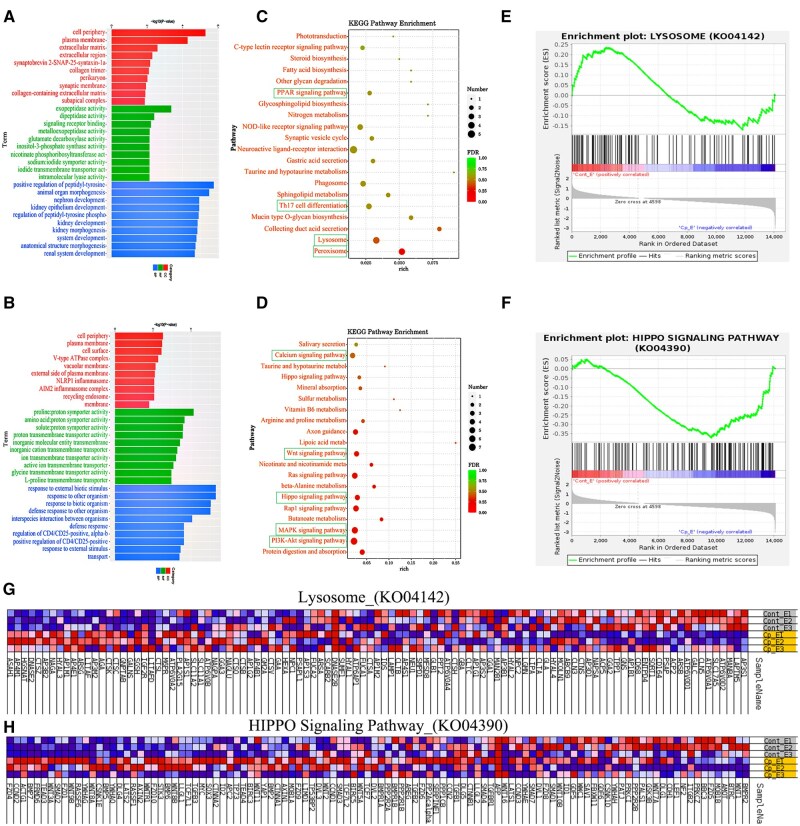
GO, KEGG, and GSEA analysis results from DEGs. GO analyzed the 10 high-ranking, highly enriched items upregulated (A) and downregulated (B) in each category. Bubble maps of the 20 high-ranking enrichment pathways upregulated (C) and downregulated (D) in KEGG analysis. GSEA analysis selected two enrichment pathways that were significantly upregulated (e) and downregulated (f) in KEGG. (G, H) Expression trends of related genes in two enrichment pathways selected by GSEA.

At the level of biological process (BP), positive regulation of peptidyl-tyrosine phosphorylation (*P *< 0.01) showed significant activation characteristics, while response to external biotic stimulus (*P *< 0.01) showed significant inhibition. Cellular component (CC) analysis showed that genes related to the cell periphery (*P *< 0.01) were significantly upregulated, while genes related to the cell surface (*P *< 0.01) were significantly downregulated. In the molecular function (MF) dimension, exopeptidase activity (*P *< 0.01) was significantly enhanced, while proline: proton symporter activity (*P *< 0.01) was significantly reduced. Further analysis showed that the related pathways of ECM organization, signaling receptor binding, and animal organ morphogenesis were significantly upregulated. In contrast, vacuolar membrane, amino acid proton symporter activity, glutathione peroxidase activity, and response to biotic organisms were significantly downregulated (Figure 3A, B).

The system analysis display was based on the KEGG pathway database. The DEGs of cryptorchidism yak epididymis and normal yak epididymis were mainly involved in five functional modules: organismal systems (40.29%), metabolism (25.18%), environmental information processing (20.86%), cellular processes (9.35%), and genetic information processing (4.32%), involving 139 significant enrichment pathways (*P *< 0.01). Twenty genes showed significantly upregulated expression patterns after analyzing the expression patterns of the 30 high-ranking core genes. At the same time, 10 genes showed significantly downregulated expression patterns. Further pathway enrichment analysis showed that upregulated genes were mainly enriched in the following key BPs: (1) steroid biosynthesis, (2) neuroactive ligand–receptor interaction, (3) regulation of Th17 cell differentiation, and (4) lysosome function-related pathways. Moreover, significantly downregulated genes were concentrated in the Wnt signaling pathway and PI3K–Akt signaling pathway, MAPK signaling pathway, and calcium signaling pathway (Figure 3C and D).

The KEGG results were independently verified using GSEA. The lysosomal function-related genes were significantly activated in cryptorchidism, while the Hippo signaling pathway genes were significantly inhibited (Figure 3E–H). The results were highly consistent with the KEGG enrichment analysis.

### Proteomic sample quality control analysis and differential protein screening

**Figure 4.**
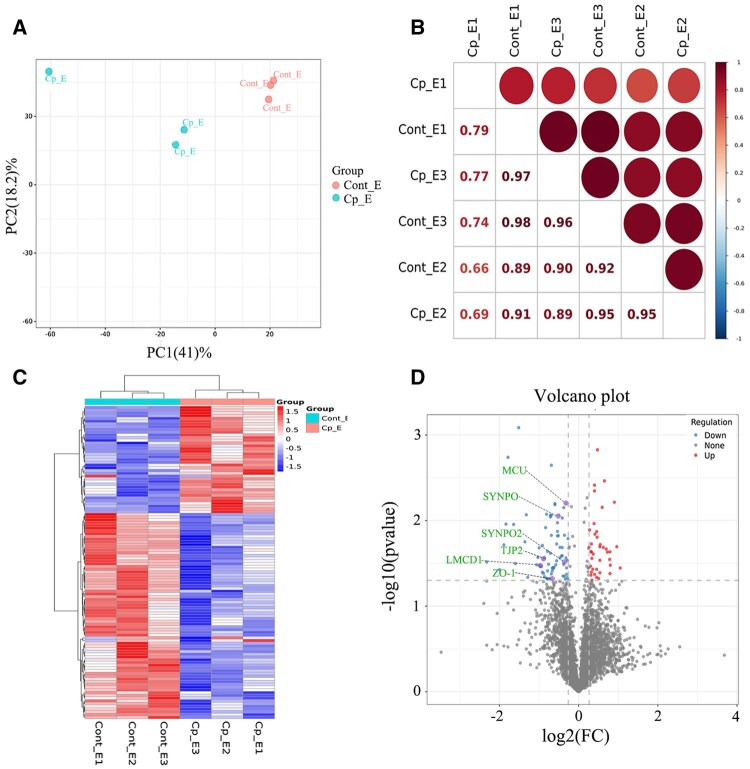
Proteomic quality control analysis of yak epididymal samples. (A) Sample principal component analysis plot. (B) Sample correlation analysis plot. (C) Cluster analysis plot of DEPs. (D) Volcano plot of DEPs, with red dots indicating highly expressed proteins in the group, blue dots indicating lowly expressed proteins in the group, and gray dots indicating non-significantly differentially expressed proteins.

**Figure 5.**
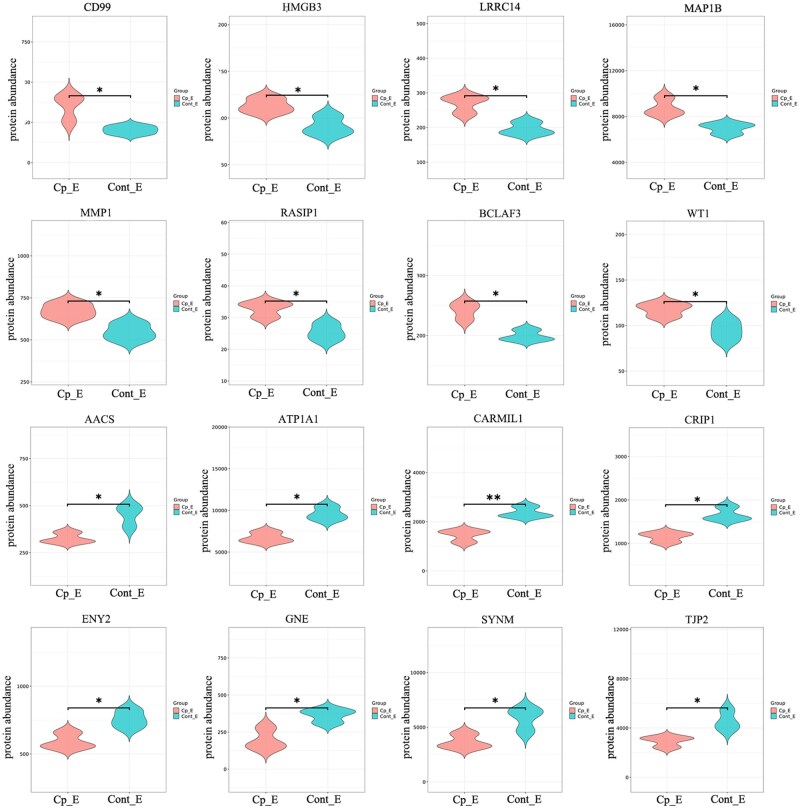
Yak differential protein violin figure. The figure shows several proteins with significant differences. The first two rows are upregulated differential proteins, and the last two rows are downregulated differential proteins. Cp_E: epididymis of cryptorchidism yak; cont_E: normal yak epididymis.

The differential protein statistical results of cryptorchidism yak epididymis and normal yak epididymis were obtained using tandem mass tags (TMT) peptide in vitro labeling quantitative technology. According to PCA analysis and sample correlation analysis, there was no aggregation between samples in different groups, and there was a good correlation between samples in the same group, indicating that the grouping was better (Figure 4A and B). The R language Pheatmap software package was used to perform a two-way cluster analysis of the union and samples of the differential genes in all comparison groups. The results showed that both groups of samples had good repeatability (Figure 4C). After screening according to credible proteins (unique peptides ≥ 1), a total of 4,074 original protein data were obtained. A total of 114 DEPs were screened in the cryptomyak epididymis and normal yak epididymis according to the protein expression difference multiple |log2FoldChange| > 1.2 and FDR-corrected *P*-value < 0.05. Of these, 39 proteins were upregulated, and 75 proteins were downregulated (Figure 4D, Supplementary Tables S3). Eight upregulated proteins and eight downregulated proteins with the most significant differences were screened by the order of *q* values from small to large (Figure 5).

### Differential protein enrichment analysis

**Figure 6.**
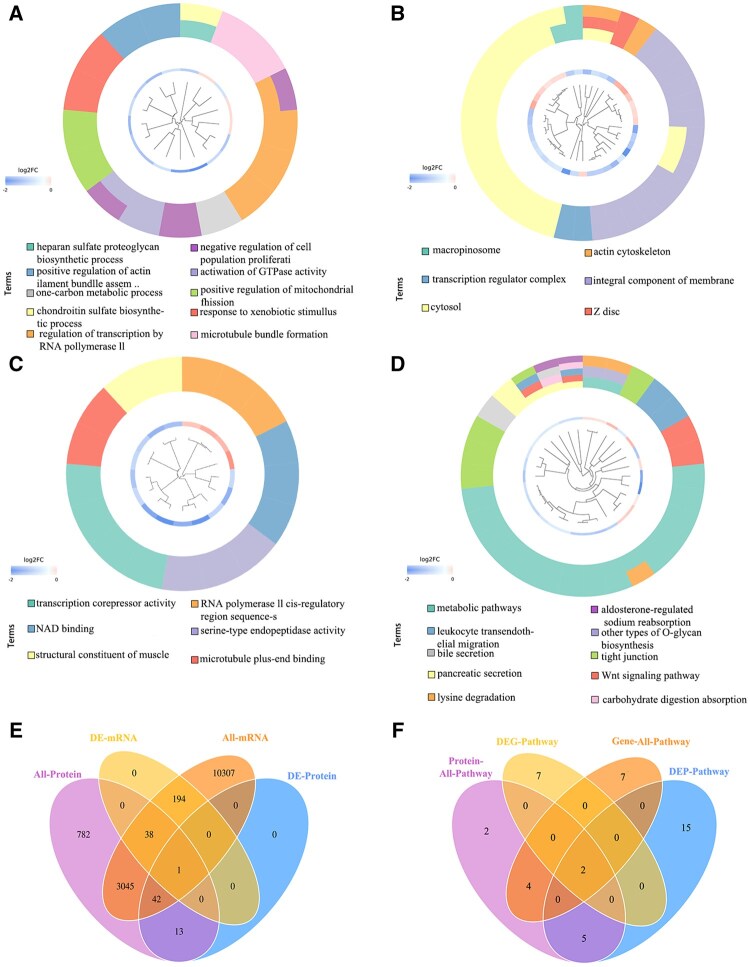
GO and KEGG analyses resulting from DEPs. (A) BP functional enrichment entries and DEPs chord cluster diagrams; (B) CC functional enrichment items and DEPs chord cluster diagrams; (C) MF functional enrichment entries and DEPs chord cluster diagrams. (D) Pathway enrichment entries and DEPs chord clustering diagram. The right semicircle of the A, B, and C graphs represents the enriched GO functional term, the right semicircle of the D graphs represents the pathway enriched term, and the left semicircle represents the differentially expressed proteins. The color ruler represents the difference multiple; the red represents the upregulated differentially expressed proteins, and the blue represents the downregulated differentially expressed proteins. (E) The number of differential genes and DEPs Venn diagram. (F) Venn diagram of the number of signaling pathways with the same enrichment trend of differential genes and DEPs.

The results of the functional significance enrichment analysis based on GO showed that DEPs showed significant enrichment in specific functional modules of cryptorchidism yak epididymis compared with the whole proteome background. By setting the significance threshold as *P *< 0.05, a total of 25 significantly enriched GO functional items were identified. Their functional distribution characteristics were as follows: In the BP dimension, DEPs were significantly enriched in the heparan sulfate proteoglycan biosynthetic process, chondroitin sulfate biosynthetic process, chondroitin sulfate biosynthetic process, activation of GTPase activity, and other critical pathways (Figure 6A). The CC analysis showed that the macropinosome, cytosol, and integral component of the membrane had a significant aggregation of associated proteins (Figure 6B). At the MF level, DEPs were specifically enriched in transcription corepressor activity, structural constituents of muscle, and NAD binding (Figure 6C).

Further pathway annotation analysis showed that DEPs were mainly involved in the following signal transduction and metabolic regulatory networks through the systematic KEGG analysis database: 1) Wnt signaling pathway, as a core pathway regulating cell polarity establishment and embryonic development; 2) leukocyte transendothelial migration, involving the molecular mechanism of immune cell infiltration; 3) dynamic regulation of tight junction affects the integrity of intercellular barrier function; 4) metabolic pathways global network, reflecting the characteristics of energy metabolism reprogramming; and 5) calcium signaling pathway is involved in the regulation of cell excitability (Figure 6D).

### Transcriptome and proteome association analysis

Transcriptome and proteome data were analyzed for the association, differential genes and differential proteins were integrated for correlation analysis, and Venn diagrams were drawn using originLab originPro (OriginLab Inc. Northampton, Massachusetts, USA). The results showed that the expression levels of one gene and one protein in the yak cryptorchidism epididymis were consistent (Figure 6E). In addition, the correlation analysis of differential gene and protein enrichment signaling pathways showed that there were two signaling pathways in the epididymis (Wnt signaling pathway and tightly connected signaling pathway) with the same enrichment trend (Figure 6F).

### Signaling pathway interaction and protein interaction network analysis

**Figure 7.**
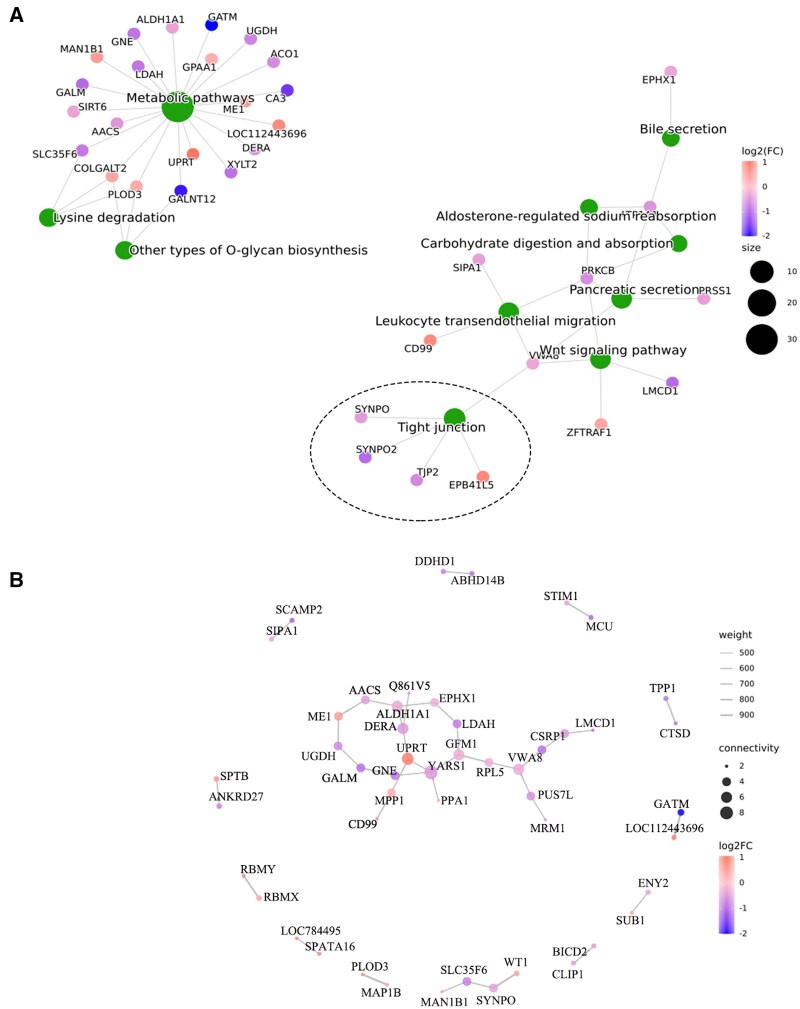
SPI and PIN maps. (A) DEPs enrichment signaling pathway association diagram. (B) The DEPs spatial interaction network diagram. Notes: Protein name abbreviations in the figure can be found in Table S3 for their full names.

The KEGG-enriched signaling pathway was used as the research object. According to the regulatory relationship between the signaling pathways, Microsoft Visio (Microsoft Corporation Redmond, Washington, USA) was used to draw the signaling pathway interaction (SPI) (Figure 7A) to obtain a significant signaling pathway regulatory relationship at the macro level. The SPI results showed that many pathways with significant enrichment of different proteins were interrelated and eventually converged together. Metabolic pathways, lysine degradation, leukocyte transendothelial migration, tight junction, and Wnt signaling pathways. The differential protein interaction network (PIN) was drawn using Igraph (University of California, San Diego, California, USA). The differential protein interaction network was composed of proteins that interacted with each other to participate in biological signal transmission, gene expression regulation, energy and material metabolism, cell cycle regulation, and other life processes. The PIN results showed that most of the screened differential proteins had spatial interaction, including some key proteins involved in the Wnt signaling pathway and tight junction signaling pathway, such as SYNPO, SYNPO2, LMCD1, and MCU (Figure 7B).

### Relative quantitative verification of differential genes and proteins

**Figure 8.**
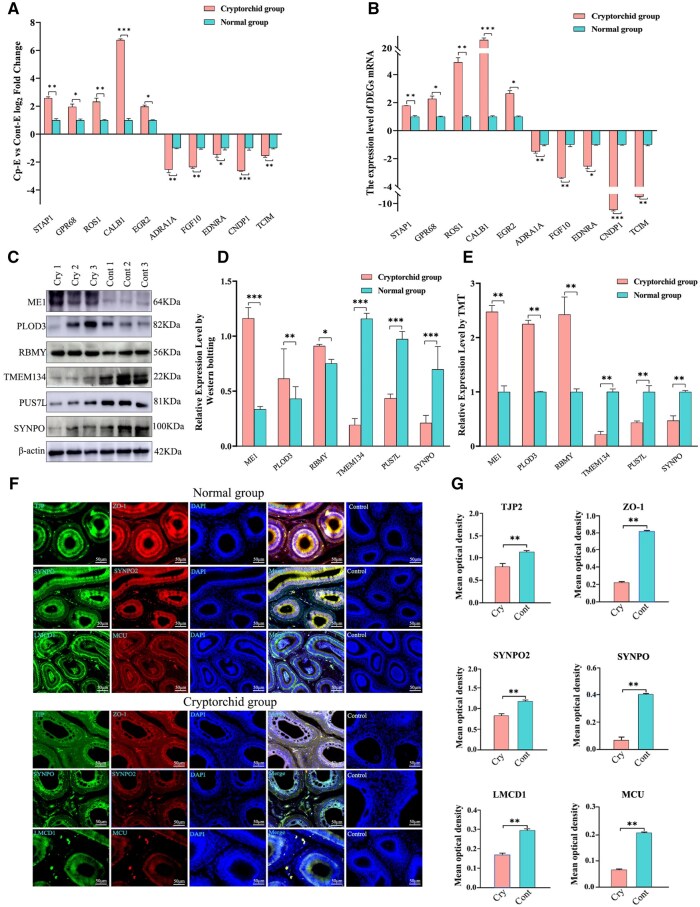
Relative quantitative verification of differential genes and proteins. (A) Quantitative results of RNAseq in the cryptorchidism group and the normal control group. (B) qRT-PCR relative quantitative results of the epididymis of the cryptorchidism group and the normal control group. (C) WB gel map of differential protein of epididymis between cryptorchidism group and normal control group. (D) Quantitative results of WB gel. (E) Quantitative results of TMT differentially expressed proteins. (F) Results of immunofluorescence localization of key proteins in the signaling pathway. (G) Results of the mean integrated optical density values of immunofluorescence images of key proteins of the signaling pathway.

This study used an orthogonal verification strategy to systematically verify RNA-seq and TMT marker quantification data and verify the biological consistency of multi-omics data. The expression of 10 randomly selected DEGs (including five significantly upregulated and five significantly downregulated genes) was verified using qRT-PCR. The quantitative results of Ct values were consistent with the results of the differential expression trends of RNA-seq (Figure 8A and B). In terms of protein level verification, WB was used to detect six DEPs (three upregulated and three downregulated), and the quantitative analysis results of gray values were consistent with the quantitative trend of TMT, indicating that the proteomic data were highly repeatable (Figure 8C–E). The subcellular localization of key proteins in the Wnt signaling pathway and the tight junction signaling pathway was further studied. The results showed that six target proteins of TJP2, ZO-1, SYNPO2, SYNPO, LMCD1, and MCU showed characteristic distribution in the epididymal tissues of the cryptorchidism group and the normal control group. These proteins were predominantly localized in the apical domain of the epithelial cells of the epididymis, the basolateral membrane, and the mesenchymal ECM (Figure 8F). Fluorescence intensity was quantified using the Image-Pro Plus image analysis system. The relative expression of all detected proteins in the cryptorchidism group was significantly lower than that in the normal control group (*P *< 0.01, Figure 8G).

## Discussion

Cryptorchidism is a common developmental disorder of the reproductive system in male mammals. Its pathological mechanism and its effect on epididymal function have always been the focus of reproductive medicine research. In this study, an integrated analysis of histology, transcriptomics, and proteomics revealed significant pathological changes and potential molecular regulatory networks in the epididymis of cryptorchidism yaks, providing a new perspective for understanding the long-term effects of cryptorchidism on epididymal function. The pathological results of this study showed that the epididymal tissue of cryptorchidism yak had significant degenerative changes, which were characterized by atrophy of the epididymal tube height, lumen stenosis, and low spermatozoa volume. This phenomenon is highly consistent with the pattern of epididymal degeneration observed in human cryptorchidism studies (Favorito et al., 2006), and similar reports have also been reported in bovine animal models (Fuke et al., 2020). The thinning of the epididymal wall may be related to the functional damage of Sertoli cells because it plays a key role in maintaining the integrity of the spermatogenic epithelium and secreting nutritional factors (Sengupta et al., 2021; Yokonishi and Capel, 2021). Quantitative analysis of EF and CF staining showed abnormal deposition of elastic fibers and collagen fibers in the cryptorchidism group, suggesting that this process may be related to the imbalance of ECM remodeling. This fibrotic phenotype interferes with the rhythmic contraction function of the epididymal duct through mechanical stress conduction (Mitsui et al., 2021), thereby affecting sperm transport and the dynamic balance of the mature microenvironment. In addition, the reduction of reticular fibers revealed using RF staining may weaken the structural coupling between the basement membrane and the stroma, leading to the loss of tissue tension and accelerating the process of epididymal atrophy. These structural abnormalities are closely related to the common low fertility in patients with cryptorchidism (Hadziselimovic and Hoecht, 2008; Holmboe et al., 2024).

Sperm maturation depends on specific proteins and ion gradients secreted by the epididymal epithelium, and disruption of basement membrane integrity may directly impair epithelial cell polarity and secretion function (Akhondi et al., 1997; Wang et al., 2021). The disturbance of ECM components observed in this study (elastic/collagen fibroplasia and reduced reticular fibers) may affect the epithelial–mesenchymal transition (EMT) through the integrin signaling pathway (M Brandão-Costa et al., 2020), leading to irreversible damage to the sperm storage microenvironment. Furthermore, immunofluorescence localization showed that the expression of key proteins ZO-1 and TJP2 in the tight junction signaling pathway was downregulated, which further supported the functional defects of the cell junction complex in the epididymis of cryptorchidism, which may aggravate the disorder of metabolite exchange during sperm maturation. At the ultrastructural level, mitochondrial swelling and ridge structure destruction directly reflect the disturbance of cellular energy metabolism caused by cryptorchidism. As the core functional unit to maintain the homeostasis of the endovascular microenvironment, the mitochondrial dysfunction of epididymis master cells may affect secretion activities through insufficient ATP synthesis (Ruan et al., 2012). At the same time, the decrease in the number of dense bodies may lead to an insufficient supply of spermatogen-related proteins CRISP1 and GPX5. Nuclear fold and chromatin marginalization suggest that the DNA damage repair mechanism may be activated, which may be related to the enhancement of local oxidative stress induced by cryptorchidism. It is worth noting that the abnormal deposition of collagen fibers under electron microscopy and the results of MS are mutually confirmed, indicating that ECM remodeling is an important feature of the pathological process of cryptorchidism. In this study, the multidimensional pathological mechanism of epididymal degeneration was systematically elucidated in the yak cryptorchidism model for the first time, including Sertoli cell dysfunction, ECM remodeling, ECM dynamic imbalance, and abnormal cell junctions. These findings provide a theoretical basis for screening therapeutic targets for cryptorchidism-related male infertility.

Transcriptome data showed that Wnt, PI3K-Akt, and calcium signaling pathways were significantly downregulated in the cryptorchid epididymis, while the lysosomal pathway was abnormally activated. Wnt signaling is the core regulator of embryonic development and tissue homeostasis (Majidinia et al., 2018; Lu et al., 2019), and its inhibition may lead to epididymal epithelial polarity disorder and abnormal lumen structure. For example, inactivation of the Wnt/β-catenin pathway can weaken intercellular adhesion by inhibiting E-cadherin expression, thereby destroying the epididymal epithelial barrier function (Xu et al., 2018). Additionally, inhibition of the PI3K-Akt pathway may aggravate tissue atrophy through a dual mechanism. On one hand, the PI3K-Akt pathway reduces the proliferation-promoting signal factor cyclin D1. On the other hand, it activates the proapoptotic factor Bax, resulting in an imbalance of epithelial cell homeostasis (Long et al., 2018; Somade et al., 2022). The enhancement of lysosome activity was specifically manifested as the upregulation of CTSB, CTSD, and other genes, suggesting that excessive activation of the autophagy-lysosome system (ALS) can accelerate the degradation of cell components (Aboelenain et al., 2015). Such components may be an important cause of the functional degradation of the epididymal epithelium. This phenomenon is consistent with reports of autophagy activation induced by oxidative stress in the testes with cryptorchidism. These results suggest that ALS imbalance may be a common mechanism of cryptorchidism pathology (Yan et al., 2024). Proteome data further supported the transcriptome findings that the downregulation of Wnt pathway-related proteins (SYNPO and LMCD1) and tight junction proteins (TJP2 and ZO-1) indicated that the function of the intercellular junction complex was impaired, which may destroy the homeostasis of the epididymal fluid microenvironment and affect the ion gradient and secretory factors required for sperm maturation. It is worth noting that the differential proteins are significantly enriched in steroid synthesis and immune regulation pathways. This result suggests that there is energy metabolism reprogramming and a chronic inflammatory microenvironment in the epididymis of cryptorchidism. These processes echo previous observations of NF-κB pathway activation of cryptorchidism in humans (Chen et al., 2012), pigs (Liu et al., 2024), and rats (Mizuno et al., 2009).

Multiomics association analysis showed that only a few DEGs showed consistent changes at the mRNA and protein levels, highlighting the key role of post-transcriptional modification in cryptorchidism pathology. For example, the abnormal glycosylation of CRISP1 may impair its ability to bind to the sperm surface (Cohen et al., 2011; Maldera et al., 2011). Additionally, the phosphorylation disorder of DEFB126 may weaken the activity of antimicrobial peptides, leading to sperm dysfunction (Tollner et al., 2008; Narciandi et al., 2016). Studies have found that the imbalance of AR expression may further aggravate the disorder of sperm maturation by inhibiting the androgen-dependent secretion function of the epididymal epithelium (e.g., inhibiting GPX5 secretion; Lareyre et al., 1997). As a key antioxidant enzyme, GPX5 is vital for protecting sperm from oxidative stress injury. Therefore, the decrease in GPX5 secretion caused by the imbalance of AR expression may further weaken the defense ability of sperm to oxidative stress, thus increasing the risk of sperm damage. The above findings suggest that cryptorchid epididymal lesions are the result of a combination of multiple regulatory mechanisms (e.g., genetic, epigenetic, and post-translational) and targeted regulation of key nodes (e.g., PI3K-Akt pathway activators or lysosomal inhibitors).

As a high-altitude adaptive species, the incidence of cryptorchidism in yaks is significantly higher than in plain cattle. Such a difference may be related to the interference of a hypoxic environment with fetal testicular descent (Yang et al., 2025). Hypoxia can inhibit the expression of INSL3, which is a key regulator of gubernaculum testis development (Duan et al., 2015; Adham et al., 2000). The significant downregulation of the INSL3 gene (transcriptome data) in this study supports this hypothesis, suggesting that high-altitude hypoxia may affect the expression of genes related to testicular descent through epigenetic regulation. In addition, oxidative stress at high altitudes may exacerbate the antioxidant defense defects of the cryptorchid epididymis, further impairing sperm motility. Oxidative stress may lead to DNA damage and affect sperm production and development, as previous studies have found (Aitken and Krausz, 2001; Ajayi and Akhigbe, 2020). In this study, although the markers of oxidative stress were not directly detected, the differential protein enrichment analysis revealed that the pathways related to oxidative stress changed significantly. This provided indirect evidence for the role of oxidative stress in the pathology of cryptorchidism and epididymis at high altitudes. Future studies can further explore the direct relationship between oxidative stress and cryptorchidism and epididymis pathology and the potential application value of antioxidant therapy in improving sperm motility.

## Conclusion

In this study, the pathological characteristics and molecular regulatory network of epididymal tissue in yak cryptorchidism were systematically analyzed using multi-omics technology. Histological analysis showed that there was abnormal deposition of elastic/collagen fibers, reticular fiber degeneration, and Sertoli cell dysfunction in the diseased epididymis, resulting in an imbalance of the sperm maturation microenvironment. Transmission electron microscopy showed ultrastructural abnormalities, such as nuclear membrane shrinkage and chromatin marginalization. The transcriptome screened 287 differential genes. Enrichment analysis revealed ECM remodeling and steroid synthesis pathway activation. At the same time, the Wnt, PI3K-Akt, and calcium signaling pathways were inhibited. Proteome identified 114 differential proteins, confirming that the expression of key regulators SYNPO, LMCD1, and tight junction protein TJP2/ZO-1 in the Wnt pathway was downregulated, and lysosomal function-related proteins were significantly upregulated. Studies have confirmed that dual regulation of the Wnt/tight junction pathway is the core mechanism of sperm processing disorders. This study constructed the molecular pathological network of cryptorchidism in yak for the first time and clarified the synergistic mechanism between Wnt/tight junction pathway imbalance and lysosomal overactivation. Additionally, this study provides a theoretical basis for the development of targeted intervention strategies. This result not only fills the gap in the study of the epididymis of cryptorchidism animals, but also opens up a new perspective for the exploration of the pathological mechanism of human cryptorchidism.

## Supplementary Material

skaf397_Supplementary_Data

## Data Availability

All RNA-seq and proteomic data used in this study are available in the SRA database (ID: PRJNA1197394, https://www.ncbi.nlm.nih.gov/sra/PRJNA1197394, accessed on 10 March 2025) and http://proteomecentral.proteomexchange.org/cgi/GetDataset? ID=PXD059203. Other raw datasets may also be requested from the corresponding author provided that all ethical requirements have been met.

## Abbreviations

ALS: autophagy-lysosome system
AR: androgen receptor
BP: biological process
CC: cellular component
CRISP1: cysteine-rich secretory protein 1
DEFB126: defensin β126
DEGs: differentially expressed genes
DEPs: differentially expressed proteins
ECM: extracellular matrix
EMT: epithelial-mesenchymal transition
ERα: estrogen receptor α
FPKM: fragments per kilobase million
GO: gene ontology
GSEA: gene set enrichment analysis
Hoxa10: homeobox protein Hox-A10
INSL3: insulin-like peptide 3
KEGG: Kyoto encyclopedia of genes and genomes
MF: molecular function
MS: Masson staining
PCA: principal component analysis
PIN: protein interaction network
PMSF: phenylmethanesulphonyl fluoride
PTMs: post-translational modifications
RFD: reticular fiber stain
RNA-seq: RNA sequencing
SPI: signaling pathway interaction
TBST: Tris-buffered saline + Tween 20
TMT: tandem mass tags
WB: western blot
